# Systematic review and single-arm meta-analysis of the clinical value of multidisciplinary team-based multimodal interventions in pulmonary nodule management

**DOI:** 10.3389/fonc.2026.1852140

**Published:** 2026-06-29

**Authors:** Dan Tong, Aimin Shao, Dan Zhu, Xianju Jiang, Jiangfeng Pan

**Affiliations:** 1 Department of Outpatient Nursing, Jinhua Municipal Central Hospital, Jinhua, Zhejiang, China; 2 Department of Respiratory and Critical Care Medicine, Jinhua Municipal Central Hospital, Jinhua, Zhejiang, China; 3 Department of Outpatient Administration, Jinhua Municipal Central Hospital, Jinhua, Zhejiang, China; 4 Department of Medical Imaging, Jinhua Municipal Central Hospital, Jinhua, Zhejiang, China

**Keywords:** clinical management, MDT, multimodal intervention, pulmonary nodule, single-arm meta-analysis

## Abstract

**Background:**

Pulmonary nodules are highly prevalent and exhibit substantial heterogeneity. Traditional single-discipline management has limitations in risk assessment and precise stratification. Multidisciplinary team (MDT)–based management, which integrates cross-specialty collaborative evaluation, has gradually emerged; however, its clinical value remains controversial and warrants comprehensive assessment through systematic review.

**Methods:**

This study was conducted in strict accordance with the PRISMA statement and was registered on PROSPERO. A systematic literature search was performed in PubMed, Embase, Web of Science, Scopus, and the Cochrane Library for studies published up to December 2025. Two independent reviewers conducted literature screening, data extraction, and quality assessment. Observational studies involving MDT-based pulmonary nodule management were included. Cohort studies were evaluated using the Newcastle–Ottawa Scale (NOS), whereas studies with diagnostic evaluation characteristics were assessed using QUADAS-2. Statistical analyses were conducted using R software (version 4.4.2). Heterogeneity was assessed using the I² test, and either fixed-effects or random-effects models were applied to pool effect sizes. Subgroup analyses were performed to explore sources of heterogeneity, sensitivity analyses were conducted using a leave-one-out approach, and publication bias was assessed via funnel plots and Egger’s test.

**Results:**

A total of 10 observational studies involving 7,658 patients were included, among which 8 studies with complete outcome data were used for the core meta-analysis. The pooled positive predictive value (PPV) of MDT-based pulmonary nodule management was 0.766 (95% CI: 0.643–0.913). The pooled guideline adherence rate was 0.766 (95% CI: 0.386–0.945). The pooled lung cancer diagnosis rate was 0.120 (95% CI: 0.053–0.210), and the pooled proportion of early-stage (I–II) lung cancer among diagnosed cases was 0.741 (95% CI: 0.422–0.918). In addition, qualitative synthesis suggested that MDT-based management may contribute to reducing unnecessary benign pulmonary nodule resection in selected clinical settings.

**Conclusion:**

MDT-based pulmonary nodule management programs were associated with relatively favorable reported outcomes in diagnostic-related metrics and guideline adherence across included studies. Further well-designed comparative studies are needed to validate these findings.

**Systematic review registration:**

https://www.crd.york.ac.uk/prospero/, identifier CRD420261295367.

## Introduction

Pulmonary nodules are defined as focal abnormal imaging opacities within the lung parenchyma with a maximum diameter of 3 cm, which can present as solitary or multiple lesions, exhibiting high heterogeneity in morphology and density (1). Comprehensive analysis based on chest CT scans shows that the overall prevalence of pulmonary nodules in screened populations is approximately 27–30%, with significant differences among populations (2); even in non-smoking populations, up to 42% of individuals have at least one detected pulmonary nodule, of which approximately 10% are clinically relevant (3). Although most nodules are benign (4), a small proportion may progress over time to primary lung cancer or other malignancies, particularly in high-risk populations such as elderly individuals or those with a smoking history, where the risk of malignant transformation is higher (5). Failure to timely identify and manage malignant nodules may not only delay the optimal window for early intervention but also allow tumor progression to advanced stages, thereby severely affecting patient survival and quality of life (6).

Traditional pulmonary nodule management primarily relies on single-discipline approaches, such as radiology or pulmonology, by analyzing imaging features and clinical information, and formulating follow-up or intervention strategies according to established guidelines, such as the Fleischner Society or BTS guidelines (7, 8). However, this single-discipline model faces multiple limitations in practice. From a diagnostic perspective, imaging evaluation is constrained by differences in specificity and sensitivity, and relying solely on radiologists’ qualitative and quantitative assessment often carries a risk of misdiagnosis or missed diagnosis, particularly for ground-glass or atypical-margin nodules, where single-dimensional interpretation may lead to inaccurate risk assessment (9, 10). In addition, conventional diagnostic workflows often insufficiently integrate clinical risk factors, which affects the precision of individualized malignancy risk stratification (11). Furthermore, for nodules requiring interventional diagnosis or minimally invasive therapy, the lack of early consensus among thoracic surgeons, interventional radiologists, and pathologists may result in diagnostic delays or unnecessary invasive procedures (12).

The multidisciplinary team (MDT) management model is a clinical approach in which specialists from pulmonology, radiology, thoracic surgery, pathology, and oncology collaboratively participate to provide comprehensive assessment and decision-making for patients (13). This model has gradually emerged in the field of pulmonary nodule diagnosis and treatment. Studies have shown that in MDT clinics or multidisciplinary nodule centers, patients are more likely to receive guideline-consistent evaluations, and management strategies can be flexibly adjusted in complex scenarios, which may facilitate more structured diagnostic evaluation and follow-up planning (14). However, some studies have pointed out that MDT implementation faces challenges regarding resource consumption and cost, and high-quality evidence demonstrating its impact on long-term clinical outcomes remains insufficient (15, 16). Given the ongoing controversy about the clinical value of MDT in pulmonary nodule management and the heterogeneity of existing studies, this study aims to systematically integrate current evidence through a single-arm meta-analysis to comprehensively evaluate the potential clinical benefits of MDT management in pulmonary nodule care.

## Methods

### Search strategy

This single-arm meta-analysis was conducted in strict accordance with the PRISMA statement and relevant meta-analysis guidelines, and was registered on PROSPERO (registration number CRD420261295367). Two researchers (Tong Dan, Shao Aimin) independently performed literature retrieval, screening, and inclusion/exclusion, with disagreements resolved by a third senior researcher (Pan Jiangfeng). The search timeframe was up to December 2025, and the databases searched included PubMed, Embase, Web of Science, Scopus, and Cochrane Library. Detailed search terms are provided in **Supplementary Material 1**.

### Inclusion and exclusion criteria

Inclusion criteria were as follows: 1) study population: patients with confirmed pulmonary nodules, without restriction on nodule type (solitary or multiple) or detection method (incidentally detected or screening-detected); 2) intervention: MDT-based management of pulmonary nodules, defined as a structured clinical decision-making model in which two or more relevant specialties participated in nodule assessment, risk stratification, follow-up planning, diagnostic decision-making, or treatment selection. Eligible MDT-related models included conventional multidisciplinary meetings, virtual MDT discussions, AI-assisted MDT assessment, guideline-based review, database-supported follow-up, or nurse-navigation-supported management, provided that multidisciplinary collaborative decision-making was a core component of the intervention; 3) study type: observational studies, including retrospective or prospective cohort studies, diagnostic evaluation studies, and case series; 4) outcome indicators: studies reporting at least one outcome, including diagnostic-related indicators, guideline adherence, lung cancer detection rate, or the proportion of early-stage lung cancer.

**Figure 1:**
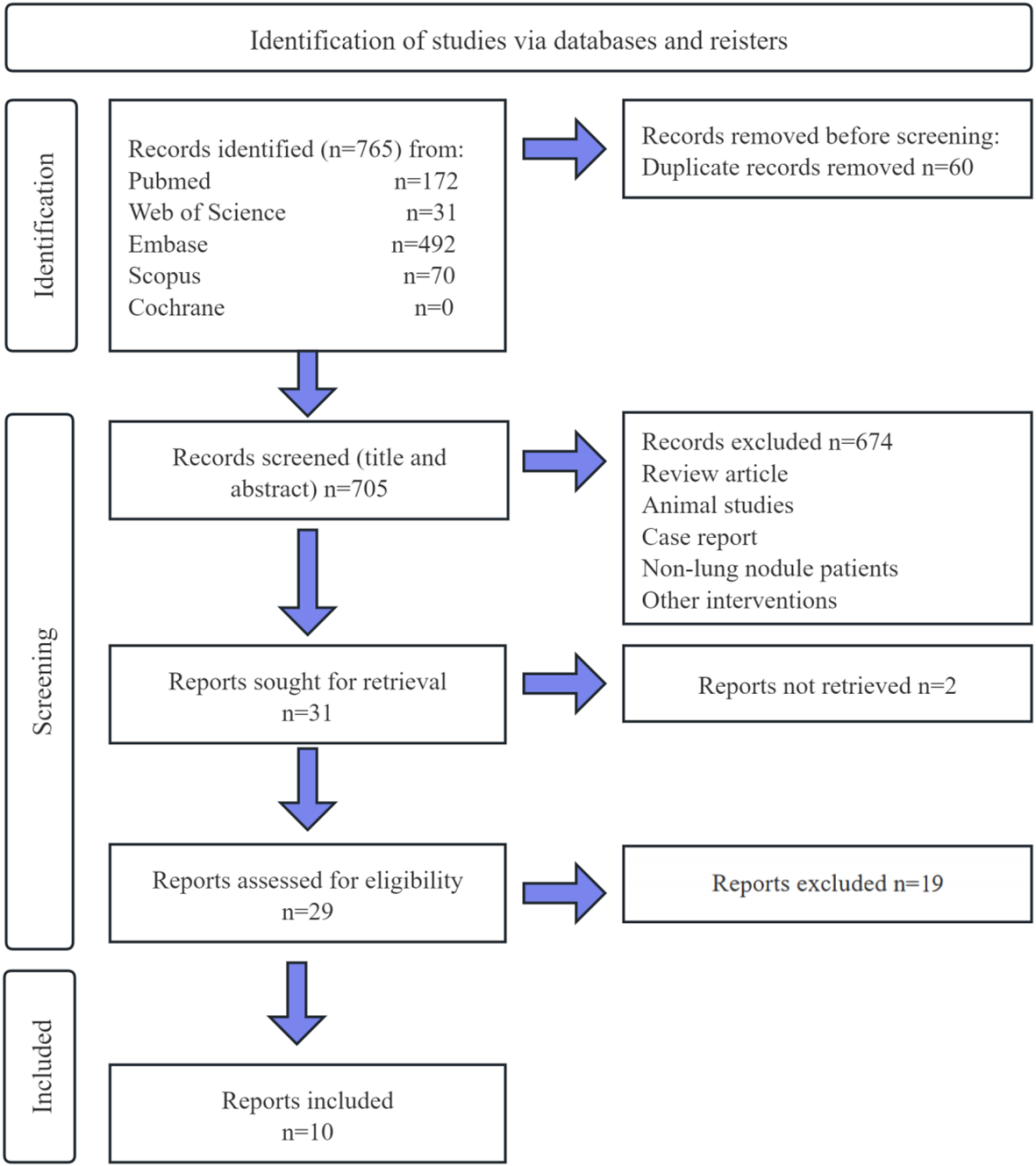
Flow diagram for the study selection process.

Exclusion criteria were as follows: conference abstracts without full texts; studies lacking patient clinical characteristics, intervention details, or outcome data; and duplicate publications with completely overlapping datasets (**Figure 1**).

### Data extraction

Based on the included studies, the following information was extracted: author, year, country, study design, study population, sample size, intervention, control intervention, and outcome indicators. Outcome indicators included diagnostic-related metrics, guideline adherence, lung cancer detection rate, and the proportion of early-stage lung cancer, among others. All data were extracted from the main text, tables, figures, or supplementary materials of the included studies, including numerical information presented in figures.

### Quality assessment

All 10 included studies were observational. To systematically evaluate the methodological quality of the included studies, NOS was used for comprehensive assessment. The NOS is an internationally recognized standardized tool for assessing the quality of observational studies, covering three major domains: first, the selection of study populations, including representativeness of exposed and non-exposed groups and comparability between groups; second, measurement of study outcomes, including the definition of outcome indicators and the completeness and reliability of data collection; third, the quality of the follow-up process, including the appropriateness of follow-up duration and follow-up rate. The total score ranges from 0 to 9, with higher scores indicating higher methodological quality. Studies with scores ≥6 are generally considered high-quality.

Quality assessment was independently conducted by two researchers, strictly following the NOS scoring criteria, with each dimension of the included studies objectively rated. Any disagreements during the scoring process were resolved by jointly reviewing the original studies and discussing thoroughly to reach consensus, ensuring the accuracy and reliability of the quality assessment.

### Statistical analysis

Statistical analyses were performed using R software (version 4.4.2). Given the lack of consistent and comparable control groups among the included studies, direct pooling of effect sizes was not feasible; therefore, a single-arm meta-analysis was conducted to combine effect sizes for the outcome indicators, comprehensively evaluating the clinical value of the interventions. Heterogeneity among studies was assessed using the I^2^ statistic before pooling effect sizes. If I^2^ < 50% and P > 0.05, indicating low heterogeneity, a fixed-effects model was used; if I^2^ ≥ 50% or P ≤ 0.05, indicating high heterogeneity, a random-effects model was applied. Subgroup analyses were conducted according to key intervention characteristics to explore sources of heterogeneity. To assess result stability, sensitivity analyses were performed using a leave-one-out approach. Publication bias was evaluated by funnel plots combined with Egger’s test, with P > 0.05 indicating no significant bias. All outcome indicators were expressed as pooled values with 95% confidence intervals (95% CI), and the significance level was set at α=0.05.

## Results

### Study selection and characteristics

A systematic search of PubMed, Embase, Web of Science, Scopus, and Cochrane Library initially identified 765 articles. After removing 60 duplicates using EndNote software, 705 articles underwent title and abstract screening. A total of 674 studies were excluded, including reviews, animal experiments, case reports, studies unrelated to pulmonary nodules, and other intervention studies. The full texts of 31 potentially eligible articles were retrieved, among which 2 articles were excluded because full texts were unavailable. Subsequently, 29 full-text articles were assessed for eligibility, and 19 studies were excluded due to incomplete data or non-conforming outcome indicators. Ultimately, 10 observational studies meeting the inclusion criteria were included. Among these, 8 studies with complete outcome data were used for the core meta-analysis. The study selection process is shown in **Figure 1**, and the detailed characteristics of the included studies are presented in **Table 1**.

**Table 1:** Basic characteristics of the included studies.

Author, Year	Country	Study design	Study population	Sample size	Intervention	Outcome indicators
Xian-Yan Liu et al. (2025) (17)	China	Retrospective diagnostic study	Pulmonary nodule patients from 3 hospitals between 2019–2020	87	Combined AI and MDT diagnosis	Diagnostic agreement: AI (κ=0.637), MDT (κ = 0.847), Combined (κ = 0.888); Sensitivity: AI (89.86%), MDT (100%), Combined (100%); Specificity: AI (77.78%), MDT (77.78%), Combined (83.33%); Accuracy: AI (87.36%), MDT (95.40%), Combined (96.55%); AUC: AI (0.838), MDT (0.889), Combined (0.917)
Gregory P. LeMense et al. (2020) (18)	USA	Retrospective cohort study	Patients with incidentally or screening-detected pulmonary nodules in Tennessee community healthcare institutions, 2016–2018	665 (Year 1), 745 (Year 2)	MDT collaboration combined with ACCP guideline review and database tracking	Proportion of Stage I–II cancers increased from 23% to 36% (Year 1) and 38% (Year 2) after program implementation; 18-month follow-up completion rate for screened patients: 71%; In Year 1, 2% of observation-waiting patients required diagnostic intervention, 1% diagnosed with cancer; Diagnostic intervention rate 27%, post-intervention confirmed malignancy rate 18.2%
Georgia Hardavella et al. (2025) (19)	Greece	Retrospective cohort study	Patients with incidentally detected pulmonary nodules at “Sotiria” Hospital, Athens, 2018–2024	2203	MDT meetings, dedicated referral forms, follow-up pathways	Discharge rate 27%; MTOS referral rate 11%; Lung cancer diagnosis rate 7%; 68% of surgically resected nodules were part-solid; VATS surgery accounted for 62.5–62.8%
Michael G. Milligan et al. (2022) (20)	USA	Prospective cohort study	Pulmonary nodule and lung cancer screening clinic patients at Massachusetts General Hospital, 2012–2019	1150	MDT multidisciplinary evaluation	Radiotherapy use in screened patients: 24.4%; 2-year local control rate: 96.2%; 2-year metastasis-free survival: 94.3%; Median age of radiotherapy patients: 73.8 years, 95.7% with smoking history
Andrew W Creamer et al. (2023) (21)	UK	Prospective cohort study	Patients with solid pulmonary nodules detected via the London SUMMIT lung cancer screening	87	MDT referral	Malignancy risk: > 200mm^3^ group 58.3%, ≤ 200mm^3^ group 13.3%; Positive predictive value: 65.9% (nodule level), 60.5% (patient level); False-negative rate 1.9%
Francys C. Verdial et al. (2020) (14)	USA	Prospective Cohort Study	Patients with incidentally detected pulmonary nodules at the Early Detection Clinic, Seattle Cancer Care Alliance, 2010–2012	113	MDT team evaluation	Guideline adherence 58%; 3-year overall survival 82–92%; No adverse events related to invasive procedures; Lung cancer diagnosis rate 29%, 88% from guideline-compliant group
D. Polanco et al. (2024) (22)	Spain	Retrospective diagnostic study	Patients with incidentally detected pulmonary nodules at virtual nodule clinic, Arnau de Vilanova University Hospital, Lleida, 2018–2019	365	Virtual MDT management	Discharge rate 43.8%; Prior follow-up guideline adherence 27.5%; Rapid lung cancer diagnostic pathway referral rate 3.6%; Survival rate of confirmed lung cancer patients 100%
Maria Lucia Madariaga et al. (2020) (16)	USA	Retrospective cohort study	Patients with incidentally or screening-detected pulmonary nodules at Massachusetts General Hospital PNLCSC, 2012–2018	747	MDT assessment, surgery/SBRT if necessary	Surgical resection rate 17.2%; Benign lesion resection rate: incidental group 20.2%, screening group 4% (P = 0.038); 5-year disease-free survival: incidental group 74.9%, screening group 89.3% (P = 0.48); 30-day postoperative mortality 0%
Thomas J. Roberts et al. (2020) (23)	USA	Retrospective cohort study	Pulmonary nodule patients at Massachusetts General Hospital PNLCSC, 2012–2019	1136	MDT multidisciplinary management, nurse navigation, smoking cessation counseling	Intervention recommendation rate 31.3% (surgery 20.5%, radiotherapy 4.8%); Guideline adherence 58%; NSCLC diagnosis rate 15%, 94.3% stage I–II; Patient adherence to recommendations 95%
Chaoyuan Liu et al. (2019) (24)	China	Retrospective cohort study	Patients with suspected malignant small solitary pulmonary nodules, Xiangya Second Hospital, Central South University, 2017–2019	360	MDT multidisciplinary discussion	Positive predictive value: MDT group 77.6%, non-MDT group 69.4% (P = 0.30); Negative predictive value (MDT group) 76.2%; Among malignant nodules, adenocarcinoma accounted for 41.4%

The 10 included studies were published between 2019 and 2025, covering China, the United States, Greece, the United Kingdom, and Spain, with a total of 7,658 pulmonary nodule patients. Interventions were centered on MDT management and combined multimodal approaches including AI-assisted diagnosis, guideline review, database tracking, virtual MDT management, nurse navigation, and smoking cessation counseling. Some studies also included traditional single-discipline management or surgery-alone interventions as control measures.

### Quality assessment

The methodological quality of the included studies was assessed according to study design characteristics. Cohort studies were evaluated using the Newcastle–Ottawa Scale (NOS), whereas studies with diagnostic evaluation characteristics were assessed using the Quality Assessment of Diagnostic Accuracy Studies-2 (QUADAS-2) tool. The NOS assessment results are presented in **Table 2**, and the QUADAS-2 assessment results are shown in **Supplementary Table 2**.

**Table 2:** Quality assessment of the included studies.

Author	Selection of population (4 points)	Comparability (2 points)	Exposure/outcome assessment (3 points)	Total score	Quality level
Gregory P. LeMense et al., 2020 (18)	3	1	2	6	Moderate quality
Georgia Hardavella et al., 2025 (19)	3	2	2	7	High quality
Andrew W Creamer et al., 2023 (21)	3	2	2	7	High quality
Michael G. Milligan et al., 2022 (20)	3	2	3	8	High quality
Thomas J. Roberts et al., 2020 (23)	3	2	2	7	High quality
Francys C. Verdial et al., 2020 (14)	3	1	2	6	Moderate quality
Maria Lucia Madariaga et al., 2020 (16)	3	2	2	7	High quality
Chaoyuan Liu et al., 2019 (24)	3	2	2	7	High quality

According to the NOS assessment, among the eight cohort studies, six were rated as high quality and two as moderate quality. Most studies achieved relatively high scores in participant selection and outcome assessment. However, because several studies were retrospective real-world investigations, their ability to control for confounding factors was limited, resulting in relatively lower scores in the comparability domain. The QUADAS-2 assessment demonstrated that the two studies with diagnostic evaluation characteristics had an overall low-to-moderate risk of bias, with the main concerns arising from the patient selection domain.

### MDT diagnostic performance evaluation

**Figure 2:**
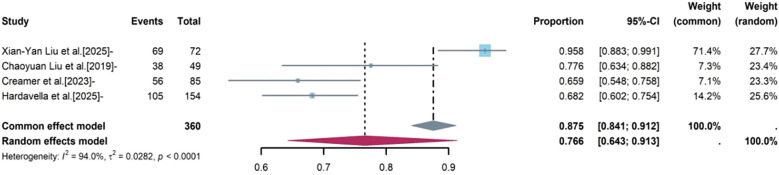
Forest plot of the meta-analysis for positive predictive value of MDT in pulmonary nodule diagnosis.

The results (**Figure 2**) showed substantial inter-study heterogeneity among the 4 included studies (I² = 94%); therefore, a random-effects model was applied. The pooled PPV of MDT-based pulmonary nodule management was 0.766 (95% CI: 0.643–0.913).

**Figure 3:**
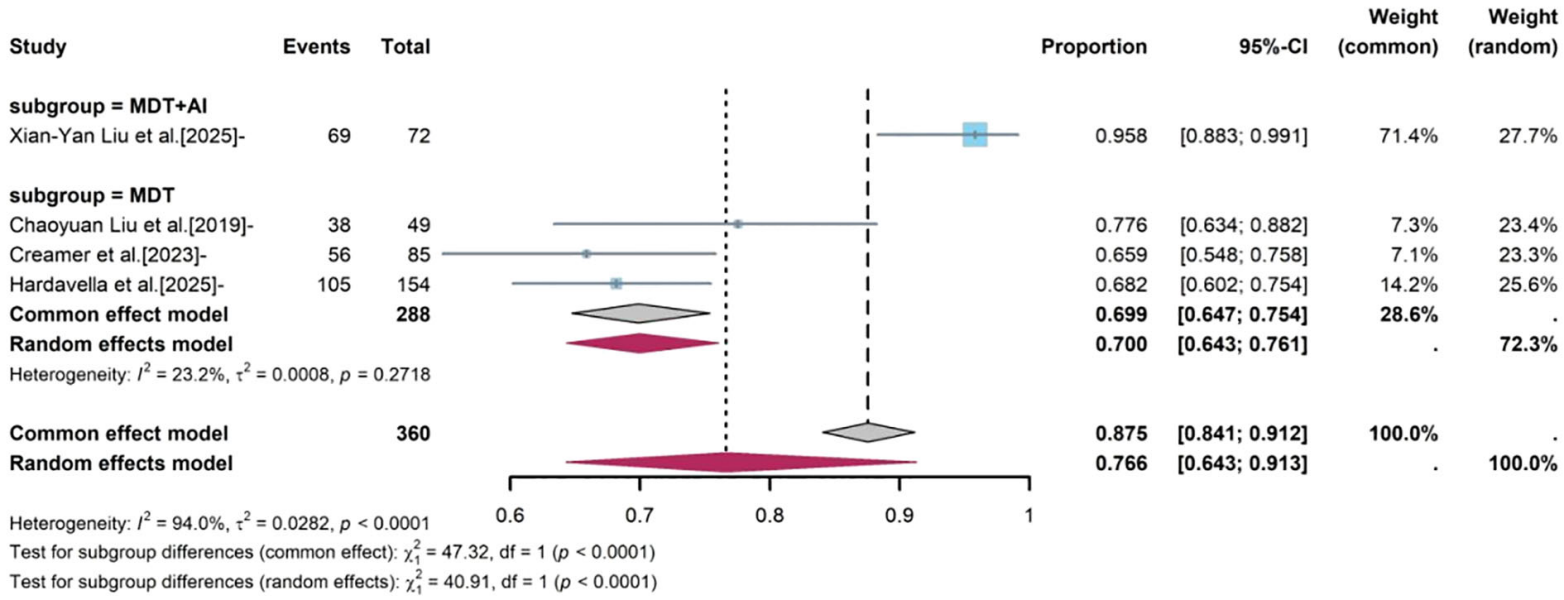
Subgroup analysis forest plot of positive predictive value for MDT in pulmonary nodule diagnosis.

Subgroup analysis based on whether AI was integrated showed that in the MDT-only subgroup, heterogeneity decreased to 23.2% (p=0.2718), with a pooled PPV of 0.700 (95% CI: 0.643–0.761); The study incorporating AI-assisted assessment reported a PPV of 0.958 (95% CI: 0.883–0.991) (**Figure 3**).

To assess the stability of MDT PPV, sensitivity analyses were performed. Sequential exclusion of individual studies did not result in substantial fluctuations of the pooled PPV, which remained at similar levels, suggesting relative stability of the pooled estimates. The funnel plot showed a roughly symmetrical distribution, and Egger’s test did not show statistically significant asymmetry (P = 0.0965) (**Supplementary Figures 1–3**).

### MDT guideline adherence

**Figure 4:**
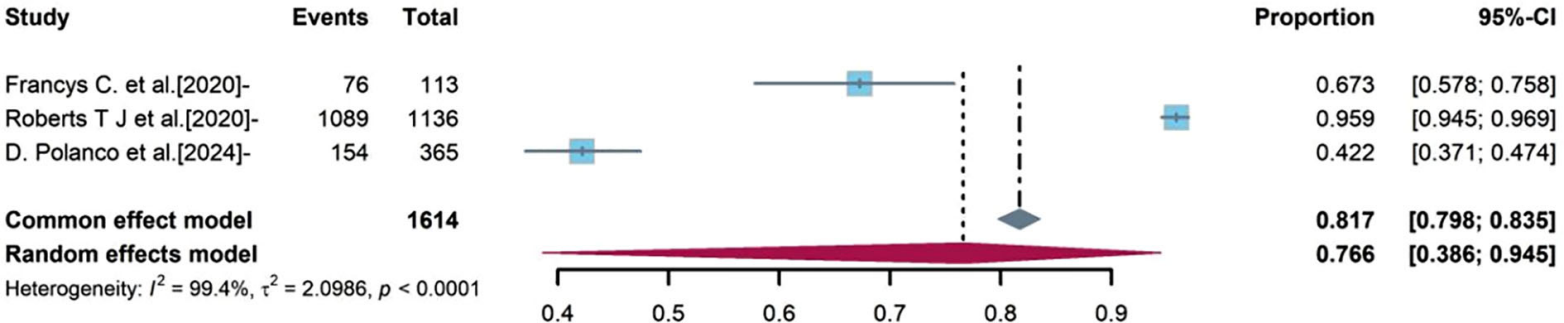
Forest plot of the meta-analysis for MDT guideline adherence in pulmonary nodule management.

Among the 3 included studies, the pooled guideline adherence rate under MDT-based pulmonary nodule management was 0.766 (95% CI: 0.386–0.945) (**Figure 4**). Sensitivity analysis showed that sequential exclusion of individual studies resulted in minimal changes in the pooled effect size, with the results remaining stable, indicating relative stability of the pooled estimates (**Supplementary Figure 4**).

### MDT and lung cancer diagnosis rate in pulmonary nodule patients

**Figure 5:**
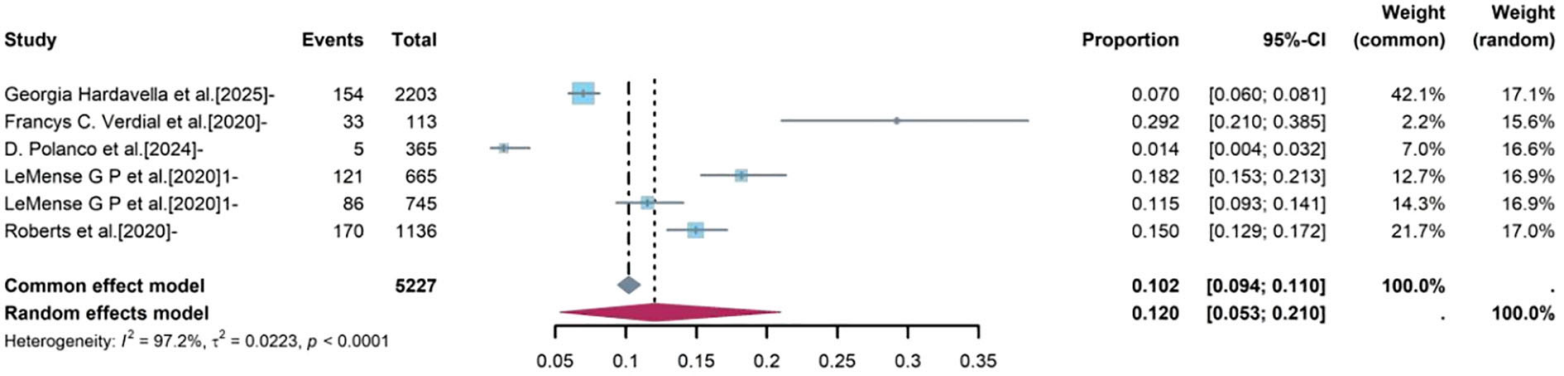
Forest plot of the meta-analysis for lung cancer diagnosis rate in pulmonary nodule patients managed by MDT.

The meta-analysis results (**Figure 5**) showed substantial heterogeneity across studies; therefore, a random-effects model was applied. The pooled lung cancer diagnosis rate among pulmonary nodule patients managed under MDT-based programs was 0.120 (95% CI: 0.053–0.210). Assessment of publication bias indicated that the funnel plot was roughly symmetrical, with no obvious asymmetry. Egger’s test did not show statistically significant asymmetry (P = 0.5540) (**Supplementary Figures 5–7**).

**Figure 6:**
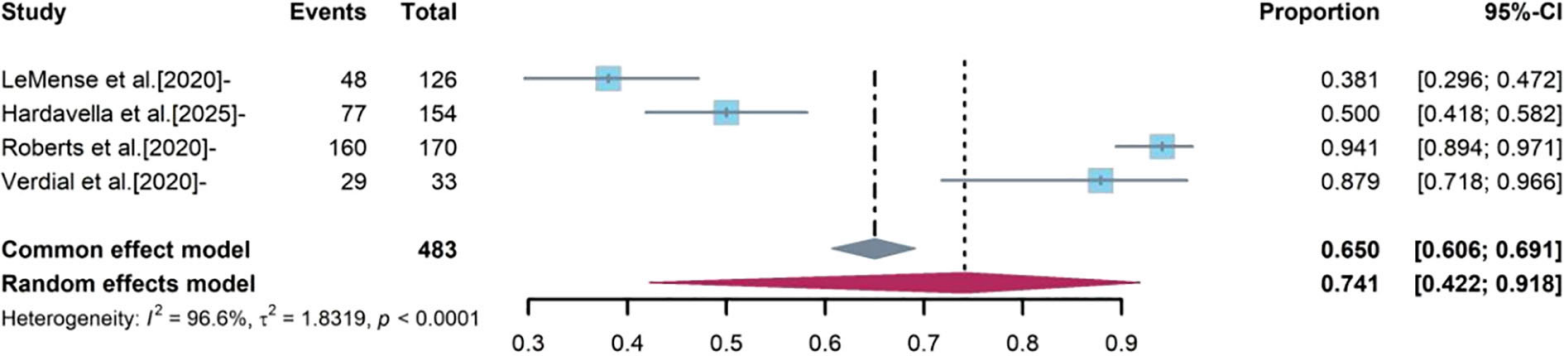
Forest plot of the meta-analysis for the proportion of early-stage (I–II) lung cancer in pulmonary nodule patients managed by MDT.

Regarding the proportion of early-stage (I – II) lung cancer among pulmonary nodule patients, the forest plot (**Figure 6**) showed high heterogeneity across studies (I^2^ = 96.6%, P<0.0001). Using a random-effects model, the pooled proportion of early-stage lung cancer under MDT management was 0.741 (95% CI: 0.422–0.918).

Subgroup analyses showed that for lung cancer diagnosis rate, stratification by guideline type did not significantly reduce heterogeneity. For the proportion of early-stage (I–II) lung cancer, stratification by nodule detection method also did not substantially reduce heterogeneity, suggesting that guideline type and detection pathway might not fully explain the observed heterogeneity(**Supplementary Figures 8**, **10**). Sensitivity analysis demonstrated that sequential exclusion of individual studies did not result in significant shifts in the pooled effect sizes, which remained relatively stable across analyses (**Supplementary Figure 9**).

### Systematic description

Some studies or outcomes were not included in the meta-analysis due to differences in data format, outcome definitions, or limited independent data sources, and were therefore summarized descriptively here.

Xian-Yan Liu et al. (2025) (17) conducted a retrospective study evaluating diagnostic performance under MDT-only and MDT combined with AI-assisted assessment models. The reported diagnostic accuracy was 95.40% for MDT alone and 96.55% for MDT combined with AI-assisted assessment. Michael G. Milligan et al. (2022) (20)conducted a prospective cohort study describing the involvement of radiation oncology within MDT management. Among pulmonary nodule patients who were screening-positive and ineligible for surgery, approximately 24.4% received stereotactic body radiotherapy (SBRT). These patients were predominantly elderly, heavy smokers with stage I lung cancer. The 2-year local control rate after treatment reached 96.3%. Maria Lucia Madariaga et al. (2020) (16)performed a single-center retrospective study, using MDT to select surgical candidates among pulmonary nodule patients. Comparing incidental nodules with screening-detected nodules, MDT evaluation was associated with a relatively low benign resection rate in screening-detected nodules; the benign resection rate in the screening group was only 4%, significantly lower than 20.2% in the incidental group, and both groups showed favorable 5-year disease-free survival.

## Discussion

This study, through systematic review and single-arm meta-analysis, comprehensively evaluated the overall clinical value of MDT combined with multimodal interventions in pulmonary nodule management. The results indicated that MDT-based pulmonary nodule management programs showed relatively favorable reported diagnostic performance and positive predictive value across included studies, with relatively high guideline adherence reported in real-world clinical practice and a relatively high proportion of diagnosed lung cancer patients identified at an early stage. These findings provide evidence for structured pulmonary nodule management.

The potential value of MDT combined with multimodal interventions primarily lies in key aspects such as nodule characterization, management pathway selection, and timing of intervention (25). Pulmonary nodule management inherently involves probabilistic decision-making, and different specialties focus on distinct aspects of the same nodule: radiology emphasizes morphological features and growth patterns, pulmonology prioritizes clinical risk factors and follow-up strategies, and surgery focuses on resectability and surgical benefit (2, 26, 27). In traditional single-discipline models, this information is often handled in isolation, potentially resulting in biased risk assessment or imbalanced decision-making. MDT enables synchronized multidisciplinary discussion, allowing nodule risk evaluation to be based on cross-validation of multidimensional information, thereby enhancing overall decision consistency and robustness. This multidisciplinary approach may be particularly valuable in structurally abnormal lungs, such as interstitial lung diseases or fibrotic lung conditions, where architectural distortion, inflammatory pseudonodules, or background parenchymal abnormalities may complicate radiological interpretation and conventional risk stratification. In such scenarios, multidisciplinary evaluation may help balance early cancer detection with avoidance of unnecessary invasive procedures (28, 29). Recent studies show that multidisciplinary discussion significantly reduces discordant decisions and repeated evaluations in nodule management, particularly in cases with atypical imaging or borderline risk (30).

Previous studies indicate that even in regions with mature guideline systems, pulmonary nodule management often suffers from insufficient follow-up, delayed intervention, and inconsistent management pathways, largely due to a lack of structured support during guideline implementation rather than guideline content itself (31). MDT incorporates structured discussion mechanisms, standardized assessment frameworks, and nodule follow-up systems, embedding guideline recommendations into routine decision-making. Under multidisciplinary management, follow-up completion rates and standardized management proportions improve significantly, while unnecessary invasive procedures and benign nodule resections decrease (32). Additionally, our findings show that lung cancers under MDT management are more concentrated at early stages; MDT allows timely diagnosis of nodules with true progression risk through rational risk stratification and follow-up scheduling, resulting in earlier staging at diagnosis (18) and higher likelihood of receiving curative treatment, thereby maximizing the survival benefit of early detection. Most included studies primarily focused on diagnostic or management-related outcomes, whereas long-term healthcare utilization, patient-centered outcomes, and cost-effectiveness remain insufficiently investigated.

The substantial heterogeneity observed across several pooled outcomes suggests limited comparability among the included studies. First, the included studies implemented MDT-based management using different organizational models, including conventional in-person MDT clinics, virtual MDT platforms, AI-assisted workflows, and management pathways combined with database tracking or nurse navigation. Variability in MDT composition, discussion frequency, decision-making processes, and resource availability across centers may have contributed to differences in reported outcomes. Second, the study populations were heterogeneous, including both screening-detected and incidentally detected pulmonary nodules, which differed in baseline malignancy risk, follow-up intensity, and clinical management strategies. Third, definitions and reporting methods for several outcome measures were not entirely consistent across studies; for example, criteria for lung cancer diagnosis rate and early-stage classification varied between studies, which may have further increased inter-study variability. In addition, several studies were conducted in specialized MDT centers with relatively mature multidisciplinary infrastructures and clinical expertise, which may limit the generalizability of the pooled findings to routine clinical practice settings. Although sensitivity analyses demonstrated relatively stable pooled estimates after sequential exclusion of individual studies, the pooled findings should still be interpreted with appropriate caution given the substantial heterogeneity and observational nature of the included evidence.

The strengths of this study lie in its systematic integration of real-world evidence on MDT application in pulmonary nodule management from multiple countries and implementation formats, including in-person MDT clinics, virtual MDT platforms, and combined database management, enhancing the external validity of the findings. Moreover, the study evaluated MDT from multiple dimensions, including diagnostic performance, guideline adherence, and lung cancer diagnostic structure, rather than focusing on a single diagnostic metric, providing a more clinically relevant assessment of overall decision-making logic. By combining single-arm meta-analysis with systematic description, this study provides integrated evidence regarding reported outcomes in MDT-based pulmonary nodule management under current real-world practice settings.

Limitations should also be noted. First, the included studies were primarily observational, lacking high-quality RCTs, and potential selection bias and confounding could not be fully excluded. Second, heterogeneity in MDT composition, discussion frequency, and intervention pathways across studies could affect outcomes and increase variability. Third, inconsistent definitions and reporting of some outcome indicators limited further pooled analysis. In addition, given the limited number of included studies in several pooled analyses, the interpretability of funnel plots and Egger’s tests may also be constrained. Finally, most studies had relatively short follow-up periods, leaving the long-term effects of MDT on survival outcomes and cost-effectiveness to be further evaluated. Prospective, multicenter studies are warranted to explore key components of MDT and their long-term clinical value.

## Conclusion

This single-arm meta-analysis summarized reported outcomes of MDT-based pulmonary nodule management programs, including guideline adherence and the proportion of early-stage lung cancer among diagnosed cases. The available evidence suggests that MDT-based management may be associated with relatively favorable clinical management outcomes in selected settings; however, substantial heterogeneity and limited comparability across studies warrant cautious interpretation of the pooled findings. Future prospective comparative studies are needed to further evaluate the impact of MDT on long-term clinical outcomes and cost-effectiveness.

## Data Availability

The original contributions presented in the study are included in the article/**Supplementary Material**. Further inquiries can be directed to the corresponding author.
