# Supplementary material for: Systematic review and single-arm meta-analysis of the clinical value of multidisciplinary team-based multimodal interventions in pulmonary nodule management
Source: Front Oncol. 2026 Jun 29;16:1852140. doi: 10.3389/fonc.2026.1852140 (PMC13382219; doi:10.3389/fonc.2026.1852140)
Supplement: Supplementary file 1 [file DataSheet1.docx]

Supplementary Table 1. Search strategy

| Database | Search terms |
| --- | --- |
| PubMed | ("Patient Care Team"[MeSH] OR Care Team* OR Patient Care Teams OR Multidisciplinary* OR Medical Care Team*  OR Health Care Team* OR Interdisciplinary* OR Healthcare Team*)AND("Multiple Pulmonary Nodules"[MeSH] OR "Multiple Pulmonary Nodule*" OR "Pulmonary Nodule*, Multiple"OR "Solitary Pulmonary Nodule"[MeSH] OR "Solitary Pulmonary Nodule*" OR "Pulmonary Coin Lesion*" OR "Solitary Lung Nodule*") |
| Web of Science | ((TS=((Patient OR Multidisciplinary OR Medical OR Health OR Interdisciplinary) NEAR/3 Care) AND TS=(Team*)) OR TS=("Care Team*") OR TS=("Healthcare Team*") OR TS=(Multidisciplinary AND (Team* OR "Health Team*"))) AND ((TS=(Multiple) AND TS=("Pulmonary Nodule*")) OR (TS=(Solitary) AND TS=(("Pulmonary Nodule*") OR ("Lung Nodule*"))) OR TS=("Pulmonary Coin Lesion*") OR TS=("Coin Lesion*" NEAR/3 Pulmonary)) |
| Embase | ('patient care team'/exp OR 'multidisciplinary team'/exp OR 'health care team'/exp OR 'interdisciplinary team'/exp OR ('patient care team*' OR 'care team*' OR 'multidisciplinary care team*' OR 'medical care team*' OR 'health care team*' OR 'healthcare team*' OR 'interdisciplinary health team*'):ti,ab,kw OR (multidisciplinary NEAR/3 (team* OR 'health team*')):ti,ab,kw) AND (('multiple pulmonary nodules'/exp OR 'multiple pulmonary nodule*':ti,ab,kw OR 'pulmonary nodule*, multiple':ti,ab,kw OR 'pulmonary nodules, multiple':ti,ab,kw) OR ('solitary pulmonary nodule'/exp OR 'solitary pulmonary nodule*':ti,ab,kw OR 'solitary lung nodule*':ti,ab,kw OR 'pulmonary coin lesion*':ti,ab,kw OR 'coin lesion*, pulmonary':ti,ab,kw)) |
| Scopus | (TITLE-ABS-KEY ( "patient care team*" OR "care team*" OR "patient care teams" OR "multidisciplinary care team*" OR "medical care team*" OR "health care team*" OR "interdisciplinary health team*" OR "healthcare team*" ) OR TITLE-ABS-KEY ( multidisciplinary W/2 ( team* OR "health team*" ) )) AND (TITLE-ABS-KEY ( "multiple pulmonary nodule*" OR "pulmonary nodule*, multiple" OR "pulmonary nodules, multiple" ) OR TITLE-ABS-KEY ( "solitary pulmonary nodule*" OR "solitary lung nodule*" OR "pulmonary coin lesion*" OR "coin lesion*, pulmonary" )) |
| Cochrane | #1 MeSH descriptor: [Patient Care Team] explode all trees  #2 (Care Team OR Patient Care Teams OR Multidisciplinary Care Team OR Multidisciplinary Care Teams OR Medical Care Team OR Medical Care Teams OR Multidisciplinary Health Team OR Multidisciplinary Health Teams OR Health Care Team OR Health Care Teams OR Interdisciplinary Health Team OR Interdisciplinary Health Teams OR Healthcare Team OR Healthcare Teams):ti,ab,kw  #3 #1 OR #2  #4 MeSH descriptor: [Multiple Pulmonary Nodules] explode all trees  #5 (Multiple Pulmonary Nodule OR Multiple Pulmonary Nodules):ti,ab,kw  #6 #4 OR #5  #7 MeSH descriptor: [Solitary Pulmonary Nodule] explode all trees  #8 (Solitary Pulmonary Nodule OR Solitary Pulmonary Nodules OR Pulmonary Coin Lesion OR Pulmonary Coin Lesions):ti,ab,kw  #9 #7 OR #8  #10 #3 AND (#6 OR #9) |

Supplementary Table 2. QUADAS‑2 Assessment of Diagnostic Studies

| Study | Patient Selection | Index Test | Reference Standard | Flow and Timing | Overall Risk |
| --- | --- | --- | --- | --- | --- |
| Xian-Yan Liu et al., 2025 | Some concerns | Low risk | Low risk | Low risk | Low-to-moderate risk |
| D. Polanco et al., 2022 | Some concerns | Low risk | Low risk | Low risk | Low-to-moderate risk |


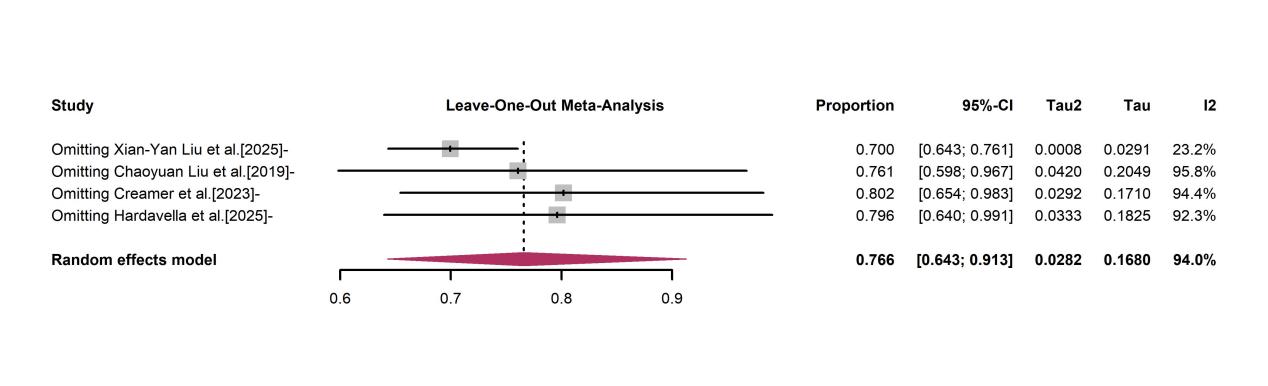


Supplementary Figure 1. Sensitivity analysis forest plot of positive predictive value for MDT in pulmonary nodule diagnosis


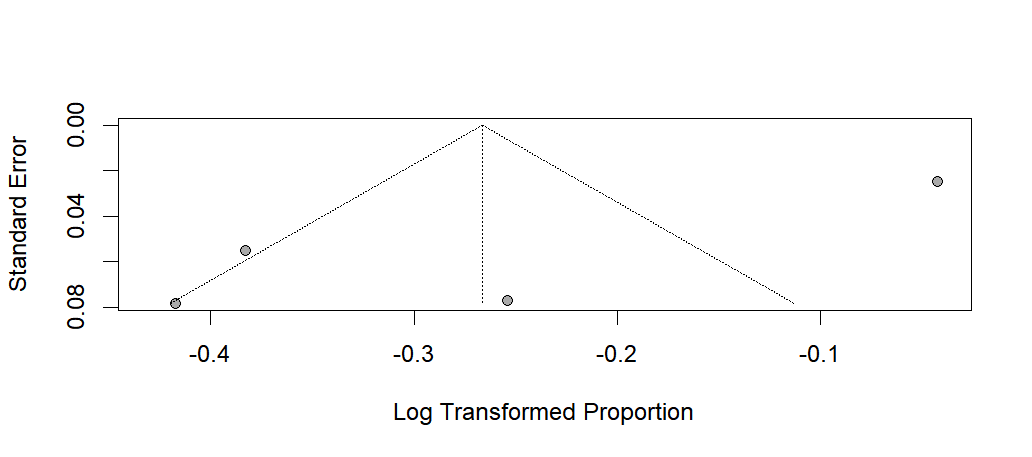


Supplementary Figure 2. Funnel plot of the meta-analysis for positive predictive value of MDT in pulmonary nodule diagnosis


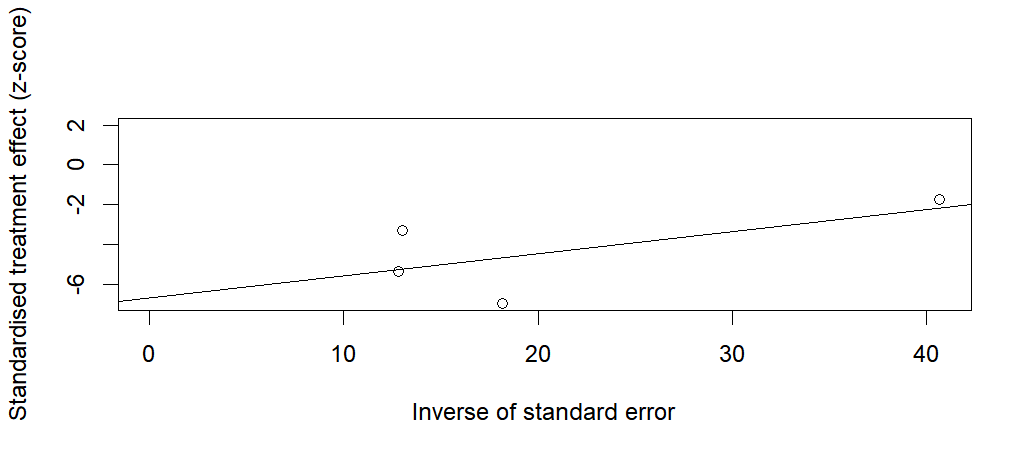


Supplementary Figure 3. Egger’s test plot for the meta-analysis of positive predictive value of MDT in pulmonary nodule diagnosis


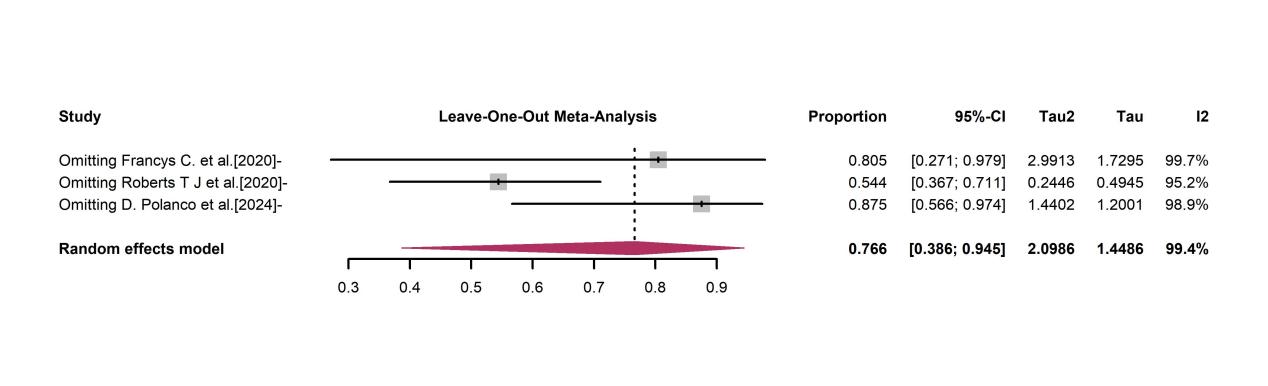


Supplementary Figure 4. Sensitivity analysis plot for MDT guideline adherence in pulmonary nodule management


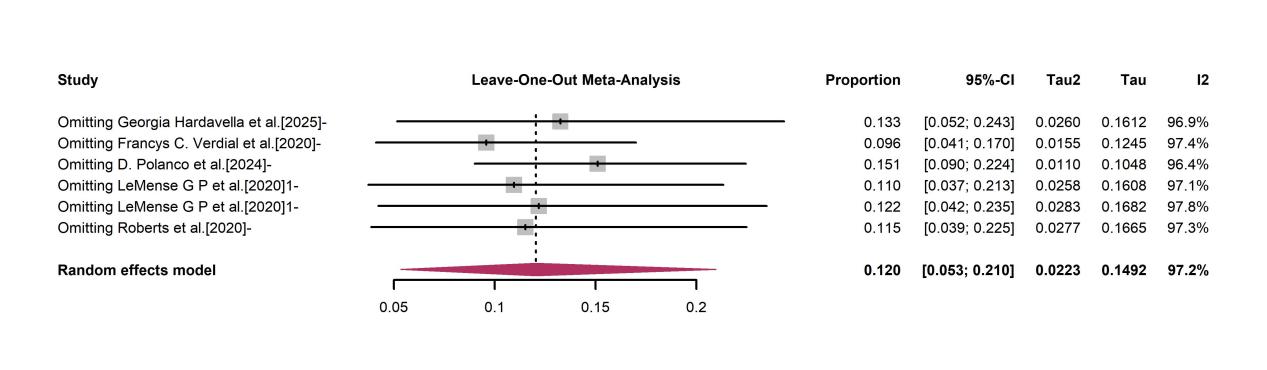


Supplementary Figure 5. Sensitivity analysis plot for lung cancer diagnosis rate in pulmonary nodule patients managed by MDT


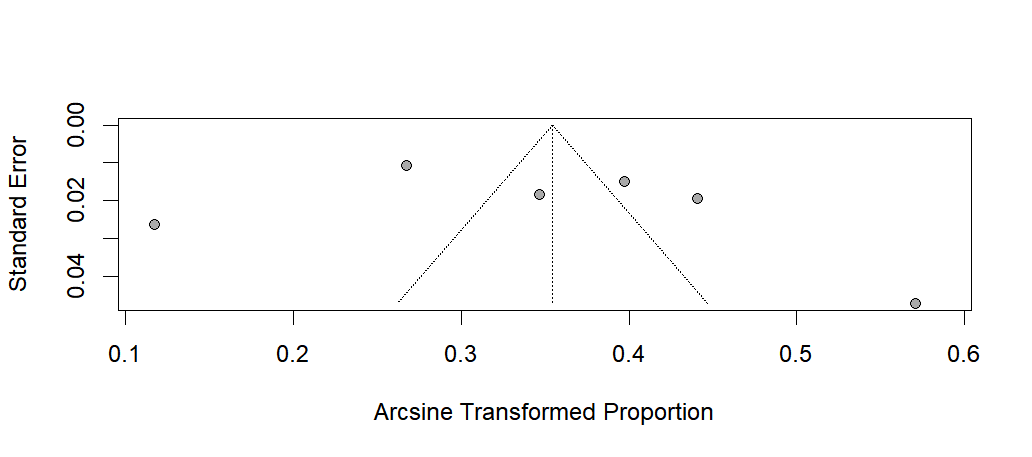


Supplementary Figure 6. Funnel plot of the meta-analysis for lung cancer diagnosis rate in pulmonary nodule patients managed by MDT


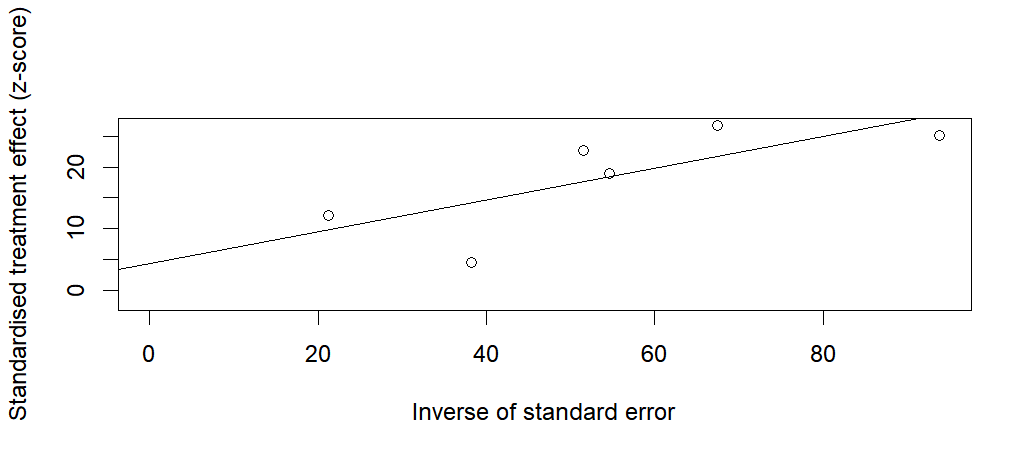


Supplementary Figure 7. Egger’s test plot for the meta-analysis of lung cancer diagnosis rate in pulmonary nodule patients managed by MDT


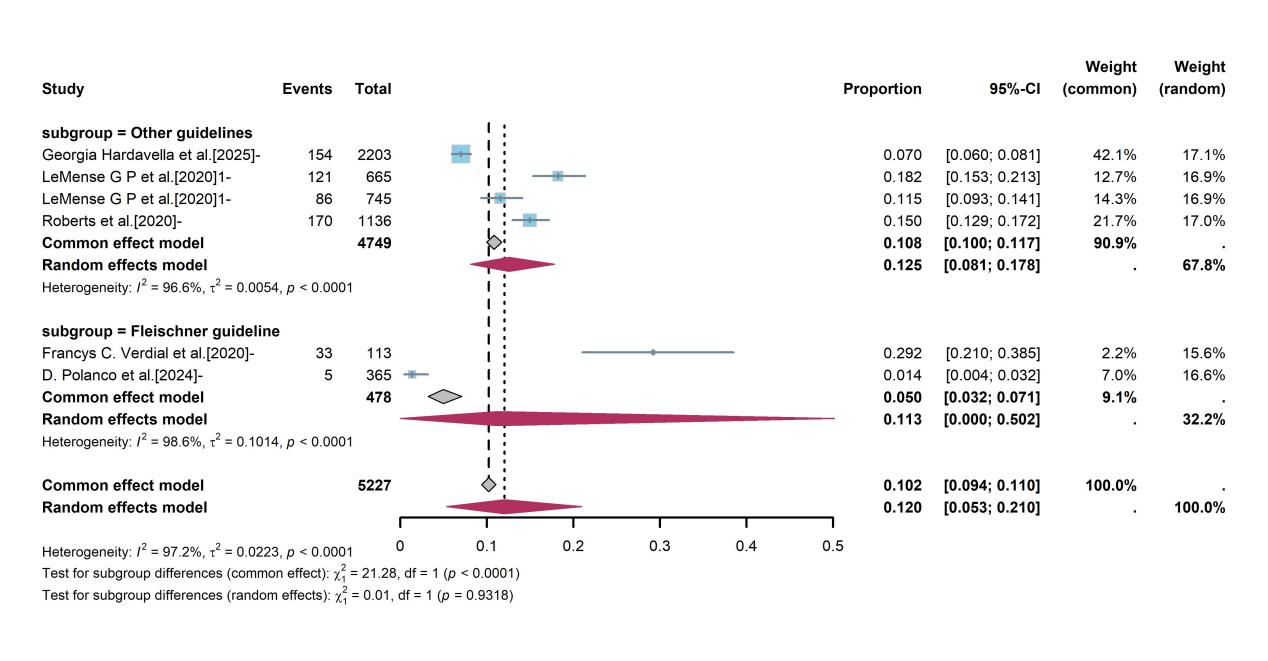


Supplementary Figure 8. Subgroup analysis forest plot of lung cancer diagnosis rate in pulmonary nodule patients managed by MDT
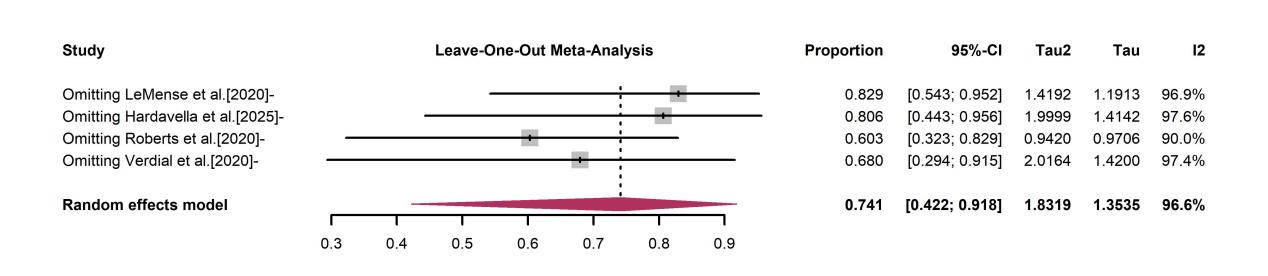


Supplementary Figure 9. Sensitivity analysis plot for the proportion of early-stage (I–II) lung cancer in pulmonary nodule patients managed by MDT


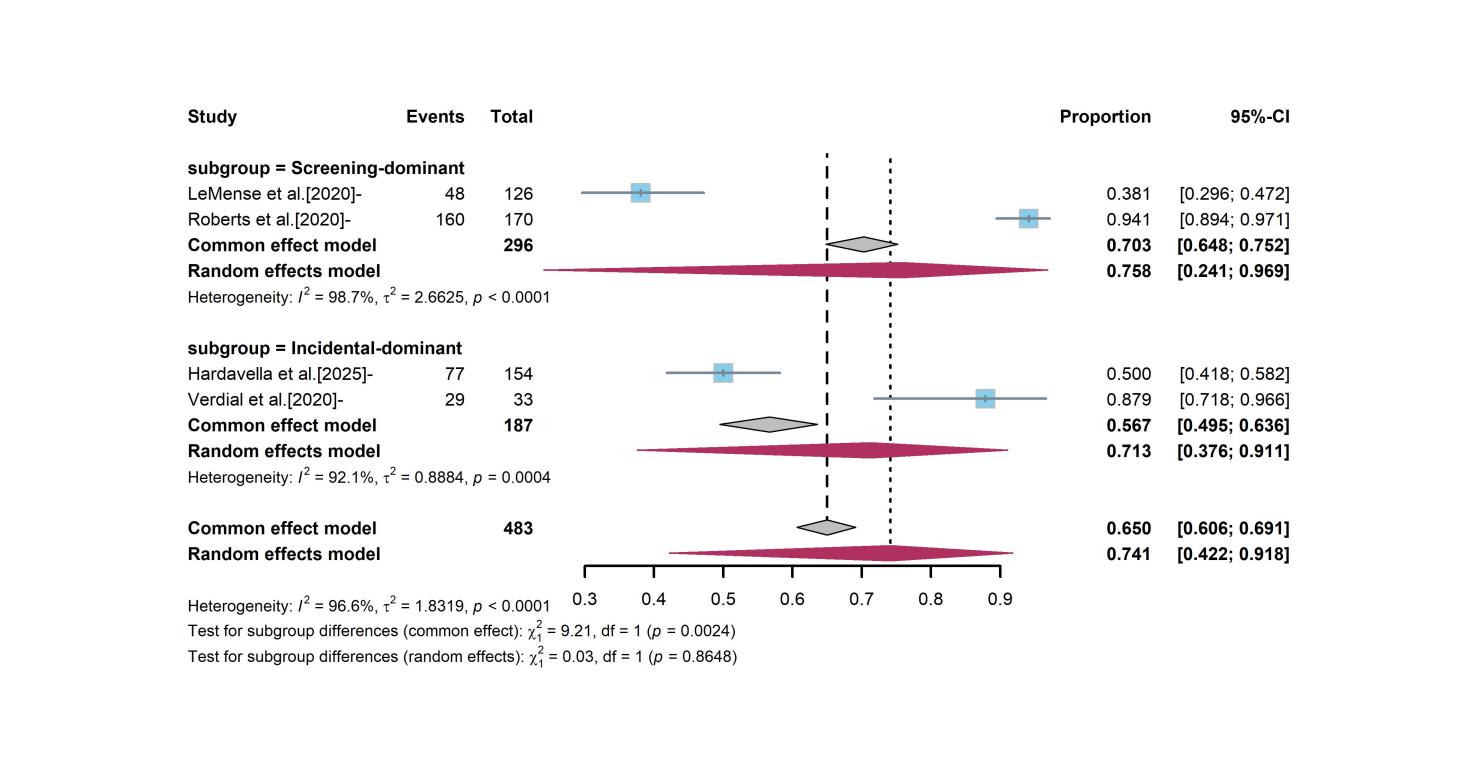


Supplementary Figure 10. Subgroup analysis forest plot for the proportion of early-stage (I–II) lung cancer in pulmonary nodule patients managed by MDT
